# Order within chaos: potential migratory strategies and individual associations in fin whales feeding off Iceland

**DOI:** 10.1186/s40462-024-00474-w

**Published:** 2024-05-09

**Authors:** Raquel García-Vernet, Diego Rita, Martine Bérubé, Julia Elgueta-Serra, Marina Pascual Guasch, Gísli Víkingsson, Marc Ruiz-Sagalés, Asunción Borrell, Alex Aguilar

**Affiliations:** 1 Department of Evolutionary Biology, Ecology and Environmental Sciences, and IRBio, Faculty of Biology, University of Barcelona, Barcelona, 08028 Spain; 2 Marine Evolution and Conservation, Groningen Institute for Evolutionary Life Sciences, University of Groningen, Nijenborgh 7, Groningen, 9747 AG The Netherlands; 3 Marine and Freshwater Research Institute, PO Box 1390, Fornubúðum 5, 220, Hafnarfjörður, Iceland; 4 Center for Coastal Studies, 5 Holway Avenue, Provincetown, MA 02657 USA; 5 Reial Acadèmia de Ciències i Arts de Barcelona (RACAB), La Rambla 115, Barcelona, 08001 Spain

**Keywords:** Baleen whale, Mysticete, Migration, Cultural transmission, Kinship

## Abstract

**Background:**

The life cycle of most baleen whales involves annual migrations from low-latitude breeding grounds to high latitude feeding grounds. In most species, these migrations are traditionally considered to be carried out according to information acquired through vertical social learning during the first months of life and made individually. However, some recent studies have suggested a more complex scenario, particularly for the species of the *Balaenoptera* genus.

**Methods:**

Here, we studied the variation of δ^15^N and δ^13^C values along the growth axis of the baleen plate from 24 fin whales feeding off western Iceland to delve into their pattern of movements and to identify potential associations between individuals. The segment of baleen plate analyzed informed about at least two complete migratory cycles. We performed cluster analyses through two different methodologies and, whenever possible, we genotyped 20 microsatellite loci to determine potential existence of kinship.

**Results:**

Results of the of δ^15^N and δ^13^C values agree with a dispersion strategy in the winter breeding grounds. However, and despite the overall large variability, several pairs or groups of individuals with no kinship showed highly similar isotopic patterns for two consecutive years for both δ^15^N and δ^13^C values.

**Conclusions:**

Our results suggest that, notably, some whales without kinship share the same migratory regime and destinations. We hypothesize that this could reflect either: (i) the sharing of particularly beneficial migratory regimes, and/or (ii) long-term association between individuals.

**Supplementary Information:**

The online version contains supplementary material available at 10.1186/s40462-024-00474-w.

## Background

In ecology, migration is defined as the seasonal movement between regions that individuals or populations carry out to obtain more favorable conditions [1]. Many taxa perform migrations and, in some species, individuals migrate in groups, influencing each other and potentially leading to new social migratory behaviors [2]. In comparison to other aspects of migration, such as departure time or migratory destinations, social factors have received limited attention [3]. This is particularly true in highly mobile oceanic species, as is the case with most baleen whales.

Most baleen whales undertake seasonal migrations with strategies that vary depending on the species and population [4]. Historically, baleen whale migrations have been considered as individual movements, alternating high-latitude feeding grounds with low-latitude breeding grounds. However, recent research has shown that the picture is more complex than previously thought, especially in the Balaenopteridae family [5, 6]. One of the most intriguing aspects is the effect of social interactions. Vertical culture, which is the transmission of cultural traditions from parents to offspring, is perhaps one of the clearest examples of this and appears to determine both migratory destinations and phenology [7–9]. Several studies show that calves learn these from their mothers during the first months of life. In some species, such as humpback whales (*Megaptera novaeangliae*) and southern right whales (*Eubalaena australis*), individuals have been found to show fidelity to the feeding and breeding grounds of their mothers, a fact that in the long-term ends up shaping the structure and genetics of populations [8, 10, 11].

In mysticetes, research on social learning and cultural transmission between non-related individuals is scarce, and most studies have been focused on humpback whales, a species in which songs and feeding techniques have been demonstrated to be transmitted horizontally to non-related conspecifics [12, 13]. In addition, several studies have reported multi-year stable associations between individuals in the feeding grounds [14–16], although long-term relationships between individuals are considered uncommon. Information about long-term social interactions in other baleen whale species, particularly in species from the *Balaenoptera* genus, is to our knowledge non-existent.

The fin whale (*Balaenoptera physalus*) is a cosmopolitan species and one of the most abundant baleen whales inhabiting the North Atlantic [17]. There is large uncertainty regarding stock structure within the North Atlantic. Genetic data do not confirm existence of structure, but diverse scientific evidences, such as dissimilarities in the response to exploitation between different localities, tagging, internal and external morphology, pollutant levels and differences in stable isotope values, among others, suggest that in this Ocean there may coexist up to seven breeding stocks of fin whales, although three of them are generally discarded because they appear incompatible with tagging results [18–22]. In the case of the Western Icelandic feeding stock, the most plausible hypothesis is that it is composed of animals breeding both in the Central North Atlantic and in the western North Atlantic [19]. This would justify finding some degree of intra-population structure in this particular area. In addition, for the other *Balaenoptera* species, little is known about social interactions occurring between individuals. In general, the fin whale is considered as non-gregarious, and the only known social relationship is that between mother and calf [17]. However, fin whales often swim in pairs and, less frequently, in larger groups [23], although the nature and duration of these associations are unknown.

Relatedness studies based on genetic markers can provide valuable insights into intra-population structure and kinship relationships. Relatedness studies have been performed in several baleen whale species and populations [24, 25], including fin whales feeding off Iceland [26]. When combined with information about migration routes inferred through other techniques, such as satellite tracking or stable isotope ratios, relatedness data offers a powerful tool to understand dynamics of cultural transmission and population structure in baleen whale communities.

On the other hand, stable isotopes provide an insight into diet, migration, and location, because their ratios in tissues reflect those from the environment in which the individual lives. They have been widely applied to investigate the migration patterns of multiple species, including marine mammals [27, 28]. From all the tissues in which stable isotopes can be determined, keratinous tissues, such as baleen plates, are particularly useful because their biochemical composition does not vary after the tissue is consolidated [29, 30]. In addition, baleen plates grow continuously, preserving a chronologically sequential record that reflects both the various environments visited by the whale and the changes that have occurred in its diet. Thanks to this, it has been possible to study variations in habitat use and diet during certain periods of the life cycle of individuals that would otherwise be impossible to monitor [31–33].

Among the various elements used for this purpose, the stable isotope ratios of nitrogen (^15^N/^14^N, from here δ^15^N values) and carbon (^13^C/^12^C, from here δ^13^C values) have received particular attention. Δ^15^N values increase between 2 and 4‰ at each trophic step, and it is typically used for inferring trophic level [34, 35]. Other factors that are known to influence δ^15^N values are spatial latitudinal distribution [36], movement between water masses [31] and nutritional condition [31, 37]. On the other hand, in marine systems, δ^13^C values increase in coastal primary producers, in comparison to primary producers from offshore areas. Therefore, δ^13^C values have been widely applied to discriminate between coastal and pelagic habitats [see for example 38]. Trophic level and latitudinal gradient also influence δ^13^C values [35, 36].

Here, we studied the variation of δ^15^N and δ^13^C values along the growth axis of baleen plates from fin whales visiting western Icelandic waters during summer. In addition, 20 microsatellites were genotyped to infer kinship relationships among the individuals. The combination of both techniques offers the opportunity to better understand the migration patterns of this stock, as well as the underlying dynamics of cultural transmission.

## Materials and methods

### Sampling

Baleen plates were obtained from 24 fin whales caught off south-western Iceland and flensed at the Hvalur H/F whaling station, Hvalfjörður, in 2013 (*n* = 5), 2015 (*n* = 9) and 2018 (*n* = 10). Sex of the individuals was determined by direct examination of their genital openings and gonads. For all individuals except one (F18030) from 2018, and for the 5 individuals from 2013, skin samples were collected from the dorsal region of the body (*n* = 18) for genetic analysis. Samples were transported from Iceland to Spain under CITES permit numbers, 15IS017MA, 18ISO36MA, ESBB00207/15I, ESBB00107/18I. After collection and during transport, baleen plates and skin samples were preserved at -20ºC.

Once at the laboratory, the baleen plates were thawed. Any adhered materials were removed with steel wool, and their surface was cleaned using a chloroform-methanol solution (2:1). Previous studies showed that gum has different δ^15^N and δ^13^C values to those of the baleen plate surface [39], so the gum was carefully removed from the plate surface with a sharp knife. Once cleaned, baleen plates were stored dry until the analysis.

The powdered samples of baleen plate tissue were extracted with a Dremel 300 series drill in 1 cm intervals, starting from the proximal end of the baleen (the most recent tissue) to the most distal (the oldest tissue), following [39]. Forty sampling points were sampled from each baleen plate, which corresponds to at least a period of two years [31, 40]).

### Stable isotope analysis

Samples (approximately 0.3 mg each) were weighed into tin capsules and combusted at 1000 °C to be analyzed by continuous-flow Isotope Ratio Mass Spectrometer, with an Elemental Analyzer (Thermo Finnigan Flash 1112 elemental analyzer; CE Elantech, Lakewood, NJ, USA), coupled to a Delta C isotope ratio mass spectrometer via a ConFlo III interface (both from ThermoFinnigan, Bremen, Germany).

International isotope secondary standards of known ^15^N/^14^N ratios in relation to the atmospheric nitrogen (air), namely ammonium sulfate (IAEA N1; δ^15^N = + 0.4‰ and IAEA N2; δ^15^N = + 20.3‰), potassium nitrate (USGS 34; δ^15^*N* = − 1.7‰), L-glutamic acid (USGS 40; δ^15^*N* = − 4.6‰) and caffeine (IAEA 600; δ^15^*N* = 1.0‰), were used to calibrate the system and compensate for any analytical drift over time. The reference materials used for the analysis were selected to ensure that the range of the reference values spanned those of the samples. All analyses were conducted at the Centres Científics i Tecnològics of the University of Barcelona (cCiT-UB).

Stable isotopes ratios are expressed following the delta (δ) notation. The relative variations of stable isotope ratios are expressed as per mil (‰) deviations from the predefined international standards according to the equation:

$${{\rm{\delta }}^{\rm{i}}}{\rm{E}}\,{\rm{ = }}\,\left( {{{\rm{R}}_{{\rm{sample}}}}\,{\rm{/}}\,{{\rm{R}}_{{\rm{standard}}}}\,{\rm{-}}\,{\rm{1}}} \right)\,{\rm{ \times }}\,{\rm{1000}}$$

In which E is the element analyzed (here, N or C) and i is the heavy isotope of that element. R_sample_ and R_standard_ are the heavy-to‐light isotope ratios (^15^N/^14^N and ^13^C/^12^C) in the sample and in the reference standard, respectively. Analytical precision for repeat measurements of the reference material, run in parallel with the baleen plate samples, was 0.3‰ for the δ^13^C and 0.1‰ for the δ^15^N values.

Samples from each year (2013, 2015 and 2018) were treated as independent datasets. All statistical analysis was performed using R (4.1.0 version).

### Time series Cluster analysis

To detect association or similarities between individuals in their δ^15^N and δ^13^C variation along the baleen plate, we applied time series cluster analysis for each dataset using two different methodologies to perform the alignment of the different baleen plates: one based on Dynamic Time Warping (DTW) and one based on Cross Correlation Functions (CCF).

#### Dynamic Time Warping (DTW) cluster analysis

DTW is an algorithm designed to find an optimal alignment between two time-dependent sequences that may contain different time-steps [41]. DTW applies a lag to each point of a series to find the best fit on the other series. Unlike the cross-correlation, DTW can apply a different lag to each point, thus accounting for changes in baleen plate growth rate, sampling errors and different morphological features of baleen plates. This technique has the advantage of providing similarity values based on the overall shape of the time-series [42]. Therefore, DTW is useful when comparing baleen plates records, especially in segments where the stable isotope ratios change fast, as is the case in fin whales [43].

Cluster analyses were performed in R (R version 4.1.0) for each dataset (i.e. whales sampled in 2013, 2015 and 2018) with the dtwclust package [44]. We used a Sakoe-Chiba window, the size of which was 10% of the length of the series [44]. Other settings were established as follows: distance measure: dtw2; centroid (prototyping function): DBA; type (clustering method): hierarchical; agglomeration method for hierarchical clustering: complete; other parameters: default. We plotted the results as dendrograms using the dendextend package.

#### Cross Correlation Functions (CCF) cluster analysis

Instead of warping the time series for finding the best alignment, as the preceding DTW algorithms do, CCF calculates the lag in which the two time series are most correlated by displacing one with respect to the other. Taking into account the lag, it measures the correlation between the two time series. This methodology is more conservative with the original shape of the time series, but it is liable of being more affected by outliers than DTW analysis. To mitigate this effect, we used an adaptation of the Manhattan distance to calculate the similarity between the aligned time series.

For each stable isotope ratio, we performed CCF to each possible pair of individuals inside each dataset, this is: 10 combinations for the 2013 dataset; 36 combinations for the 2015 dataset, and 45 combinations for the 2018 dataset. To avoid the comparison of tissue synthetized during different seasons, we fixed a maximum lag of ± 3 centimeters. We stored the optimum lag for each pair of baleen plates.

Dissimilarity between each pair of individuals was calculated using an adaptation of Manhattan distance:

$$\eqalign{& Dissimilarity\left( {adapted\,Manhattan\,distance} \right) \cr & = {{\sum\limits_1^n {|{\delta ^i}{E_{jk}}} - {\delta ^i}{E_{z\left( {k + lag} \right)}}|} \over n} \cr}$$

Where j and z are two individuals from the same dataset, k is the sample position, n is the total number of samples being compared, and lag means the optimum lag estimated for the “jz” pair before performing the data extraction.

We estimated the robustness of the results using a jackknife-like technique. 1% of the values in each dataset (4 values for 2019 and 2015 and 2 values for 2013) were randomly extracted and the dissimilarity index was again estimated for all the possible pairs. These dissimilarity measures were used to perform a second clustering analysis using the function hclust with the default parameters. This process was repeated 10.000 times for each dataset and stored all produced models as a list of “phylo” objects.

We used the ape package [45] to obtain a consensus dendrogram using the function “consensus”. The proportion for a clade to be represented in the consensus tree was fixed as 0.95 (*p* = 0.95). We used the function “prop.clade”, which counted the number of times that bipartitions were present in all the trees computed, as an approximation of the clades support. We plotted the results as dendrograms using the dendextend package.

#### Adaptation of Manhattan distance – visualization

Finally, for each stable isotope, we visualized the results of the adapted Manhattan distance, used as dissimilarity measure in the CCF clustering. We calculated the dissimilarity for each pair of individuals of the original dataset without extracting any datapoint, and performed a cluster analysis using the function hclust with default parameters.

We plotted the cluster results, together with its dissimilarity measures, by using the function heatmap. We applied a row scaling to improve the visualization. Row scaling does not play a role in the clustering analysis but only serves to display the colors representing the dissimilarity measures between pairs. To finish, we printed a list with each individual and the corresponding pairs that showed lower dissimilarity values.

### Visual exploration

We considered that two or more whales were likely to either: (i) have same migratory strategy and/or (ii) be associated, when they met the following requirements:

i) Individuals were grouped together by the DTW cluster and the CCF cluster analyses with δ^15^N and/or δ^13^C values.
ii) Individuals presented a low dissimilarity measure in the CCF clustering when all datapoints are analyzed (Material and Methods 2.3.3.) compared with their other possible pairs (top 1 in case of the 2013 dataset, top 2 in case of the 2015 and 2018 dataset) for at least one stable isotope ratio.
iii) Lags were independently determined during the cluster analyses for δ^15^N and δ^13^C values. Small differences (up to 1–2 centimeters) could be attributed to metabolic factors, such as differences between δ^15^N and δ^13^C turnover rates [46, 47]. However, larger lag differences probably reflect variations in migration timing. Therefore, individuals in which the lags for δ^15^N and δ^13^C values were > 2 were considered candidates of sharing a common migratory strategy, but their potential association was discarded.

For all pairs of whales that met the 2 first requirements for at least one stable isotope ratio, we visually explored the similarities between these individuals by plotting their raw data with their best alignment inferred through cross-correlation functions. We also plotted the groups of whales that met the first requirement for at least one stable isotope ratio. The third requirement was considered for interpreting the nature of the potential associations across individuals.

### Genetic analysis

#### Laboratory analyses

For individuals for whom skin samples were available, total-cell DNA was extracted from tissue samples either by standard phenol: chloroform extractions [48] or using the Qiagen dNeasy™ Blood and Tissue Kit (Qiagen Inc.) according to the manufacturer’s instructions. Extracted DNA was re-suspended in 1x TE buffer (10mM Tris-HCl, 1mM EDTA, pH 8.0).

The genotypes were determined at 20 microsatellite loci, using the following oligonucleotide primers: EV001, EV037 and EV094 [49], GATA028, GATA098, GATA417, and TAA023 [50], GT011 [51], GT023, GT211, GT271, GT310, and GT575 [52], AC087 and CA234 [53], GATA25072, GATA43950, GATA5947654, GATA6063318, GATA91083 [54]. Samples were genotyped in multiplex PCR reactions, using the MM2X™ Multiplex kit Plus (Qiagen Inc.) in 5µL reaction volumes. The thermos-cycling conditions were: 2 min. at 94 °C, followed by 35 cycles at 94 °C (30 s.), 57 °C (90 s.) and 72 °C (30 s.) followed by a final cycle at 68 °C (10 min.). The PCR products were separated by capillary electrophoresis using an ABI 3730 (Applied Biosystems ABI Prism™ 3730). The size of the amplification products was estimated using the Genescan ROX-500 size standard (Applied Biosystems Inc.) and GENEMAPPER™ (v. 4.0; Applied Biosystems Inc.).

#### Relatedness analysis

Allele frequencies, observed and expected heterozygosity as well as the probability of identity for each locus was estimated using the software GIMLET v. 1.3.3 [55].

Relatedness between individuals was identified using the maximum likelihood approach implemented in ML-RELATE [56]. This software can accommodate for the presence of null allele, which is estimated using the Hardy-Weinberg test for excess homozygotes. If null alleles are present, they are then specified, and all following calculations use the maximum likelihood estimates of the frequency of the null alleles.

#### Relationship analysis

The relationship (i.e., unrelated, half-sibling, full-sibling, parent-offspring) between pairs of individuals was estimated using ML-relate. ML-relate indicates the relationship) with the highest likelihoodIL(R) and specifies how much lower the log-likelihood Delta Ln(L) are for the other relationships. The level of confidence was estimated with levels of confidence of 0.75, 0.50 and 0.25.

For the individuals with similar stable isotopes profiles, we then statistically assessed the reliability of our results by performing a “specific hypothesis test of relationship”, implemented in ML-relate, that attempted to exclude an alternative relationship (here “Unrelated”) by performing 50,000 simulations. We considered that the relationship was much more likely than “Unrelated” when *p*-values were lower than 0.05 [56].

## Results

The total number of samples analyzed from the 24 fin whale baleen plates was 960. Mean and standard deviations of δ^15^N and δ^13^C values for each individual are detailed in Table S1. Results of the δ^15^N and δ^13^C patterns of individuals are shown in Figures S1 and S2, before performing any alignment or correction. δ^15^N values showed higher variability and clearer oscillations than δ^13^C values.

### Time series Cluster analysis– DTW and CCF clustering

**Fig. 1:**
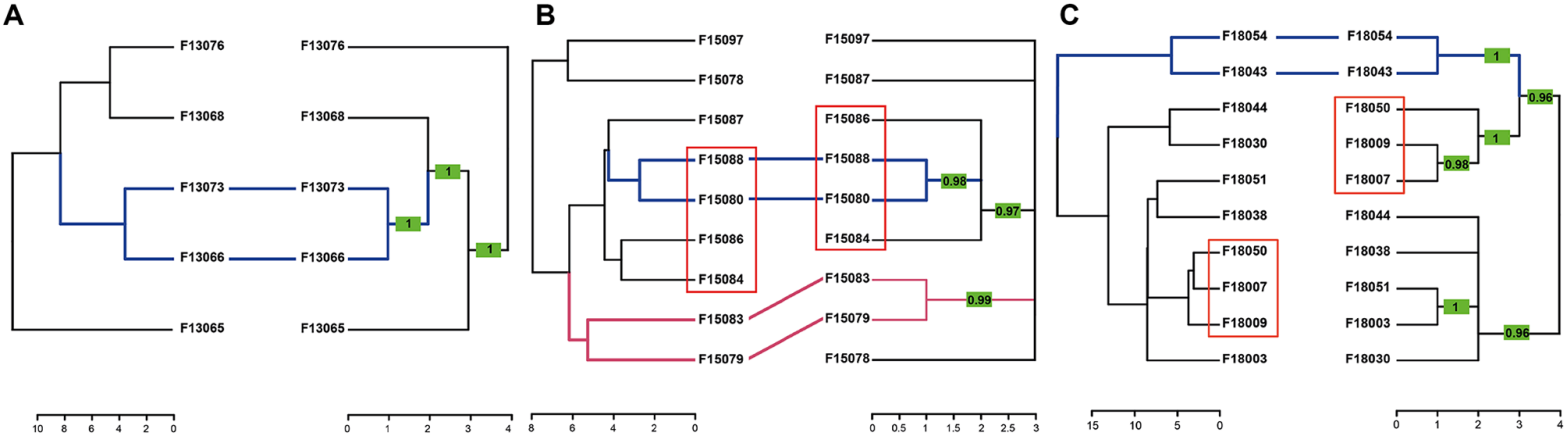
Dendrograms showing the results for δ^15^N cluster analyses for 2013 (**A**), 2015 (**B**) and 2018 (**C**) datasets. Results from the DTW (dynamic time warping) clustering are presented at the left dendrogram of each panel, and results from the CCF (cross correlation functions) clustering are presented at the right dendogram. Colored lines show common branches and groups for both methodologies. Red squares show groups that were composed by the same individuals, although the internal structure was not maintained. Finally, in the CCF clustering, branches in which *p* > 0.95 are indicated inside green boxes

Results from the DTW and the CCF hierarchical clustering performed using δ^15^N values are shown in Fig. 1, while results using δ^13^C values are shown in Figure S3. The topology of dendrograms differed between the two methods except for the clustering performed using the δ^13^C value of the 2013 dataset. Some CCF dendrograms presented polytomies, which corresponded to the clades with *p* < 0.95. Although we found large variability between individuals, especially for the δ^15^N values (Figure S1), several pairs or small groups of individuals were consistently grouped together, regardless of the clustering method.

Dendrograms for the 2013 dataset using δ^15^N values showed one common pair, composed by the individuals F13073 (male) and F13066 (male). When performing the same clustering but with the δ^13^C values as input, we found the same pair (F13073 and F13066) consistently grouped by both methods. In addition, δ^13^C clustering also showed another pair composed by individuals F13076 (male) and F13068 (female), which was also observed in one δ^15^N clustering analyses (DTW).

Dendrograms for the 2015 dataset differed in their general topology. When using δ^15^N values, two pairs were common between the two methodologies: F15080 – F15088 (male, male) and F15083 – F15079 (female, female). In CCF cluster analysis, only three dendrogram clades were well supported: those of the aforementioned pairs, and the cluster with individuals F15080, F15088, F15084 and F15086 (two males and two females), that were also grouped in the DTW cluster (Fig. 1B). Clustering performed with δ^13^C values showed low concordance between both methods, being only one pair consistently grouped: F15086 (female) and F15083 (female – associated with F15079 in δ^15^N clustering).

Finally, dendrograms for the 2018 dataset also presented differences in the general topology. We only found one common pair in both dendrograms for the δ^15^N analyses, composed of individuals F18054 (female) and F18043 (female). All the remaining tree topologies differed between methodologies, although we found a consistent group of three individuals (F18050, F18007, F18009, one female and two males). When using δ^13^C values, the general topology differed compared to the δ^15^N values. We consistently found the same pair (F18054 and F18043), but the calculated lag was 4 centimeters different to the optimal for δ^15^N values. Results of the δ^13^C clustering also showed two additional pairs, F18030 (female) and F18050 (female – associated with F18007 and F18009 in the δ^15^N analyses), and F18044 (female) and F18003 (male).

### Adaptation of Manhattan distance – visualization

**Fig. 2:**
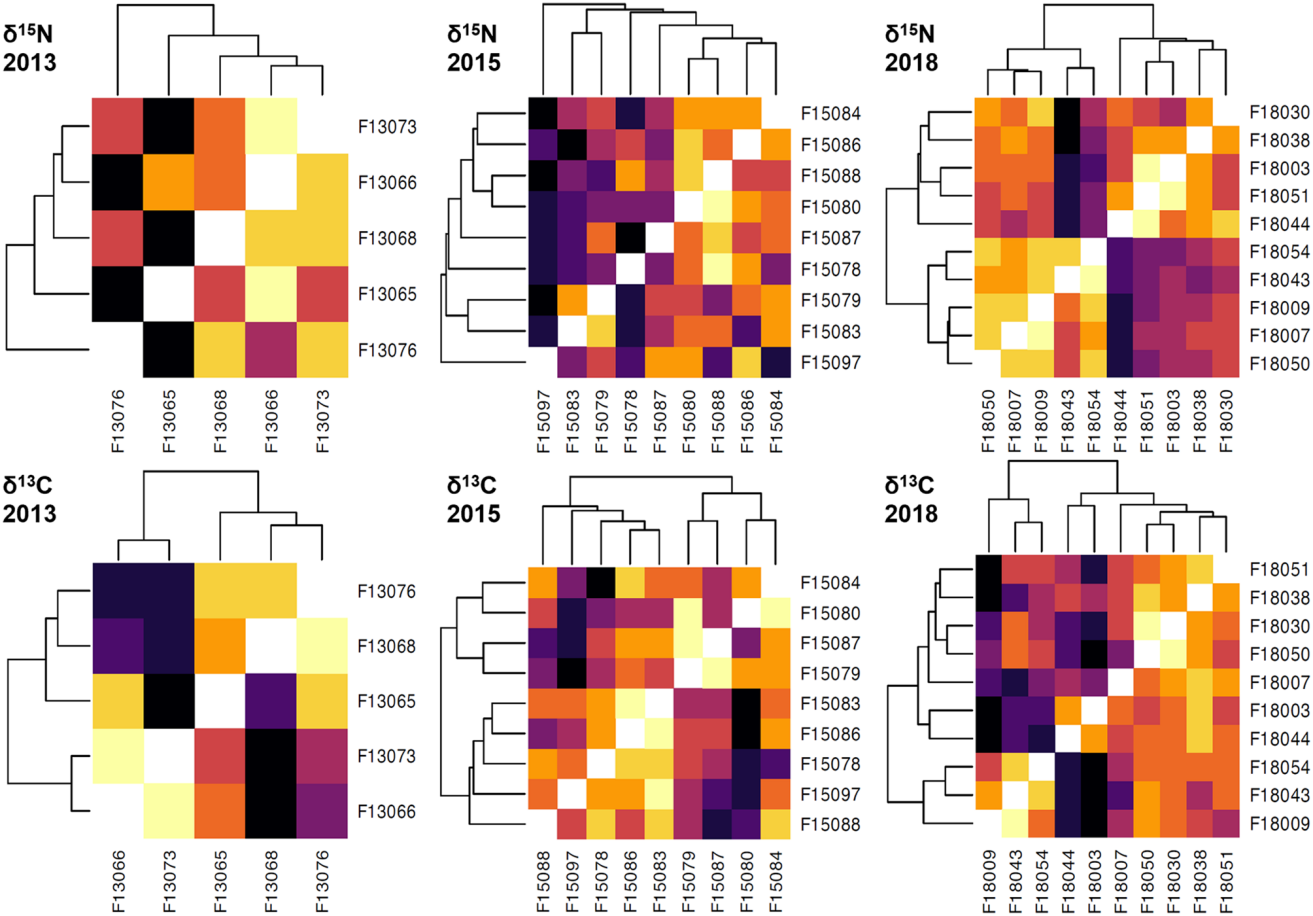
Heatmap showing the results of the CCF (cross correlation functions) clustering and its dissimilarity values (based on the adapted version of the Manhattan distance), for 2013, 2015 and 2018 datasets. CCF clusters were performed using all the data of each dataset, without extracting any point. Colors of the heatmap are indicative of the dissimilarity values between each pair of whales. Lighter colors indicate higher similarity between individuals

To visualize the dissimilarity between each pair of individuals, we plotted our results using the heatmap function in R, and we printed a list with the individuals and the corresponding pairs that showed lower dissimilarity values, i.e. more similarity (Fig. 2, Table S2).

In general, we observed a great variability among individuals for both δ^15^N and δ^13^C values, without any clear group structure apart from the similarities between specific individuals. This result was in concordance with the low supports obtained in the CCF dendrograms topologies (Fig. 1, Figure S3). One exception was found in the 2018 dataset when analyzing δ^15^N values (which corresponds with the best supported dendrogram of the CCF clusters), where we found a group of whales with low dissimilarity measures among all the individuals. The group (showed in Fig. 2C; yellowish lower left quarter) was composed of whales F18050, F18007, F18009 (which were also grouped in the previous analyses) and F18043 and F18054 (which were grouped as a pair in the previous analyses). However, this group was not detected when clustering the δ^13^C values.

### Visual exploration

**Fig. 3:**
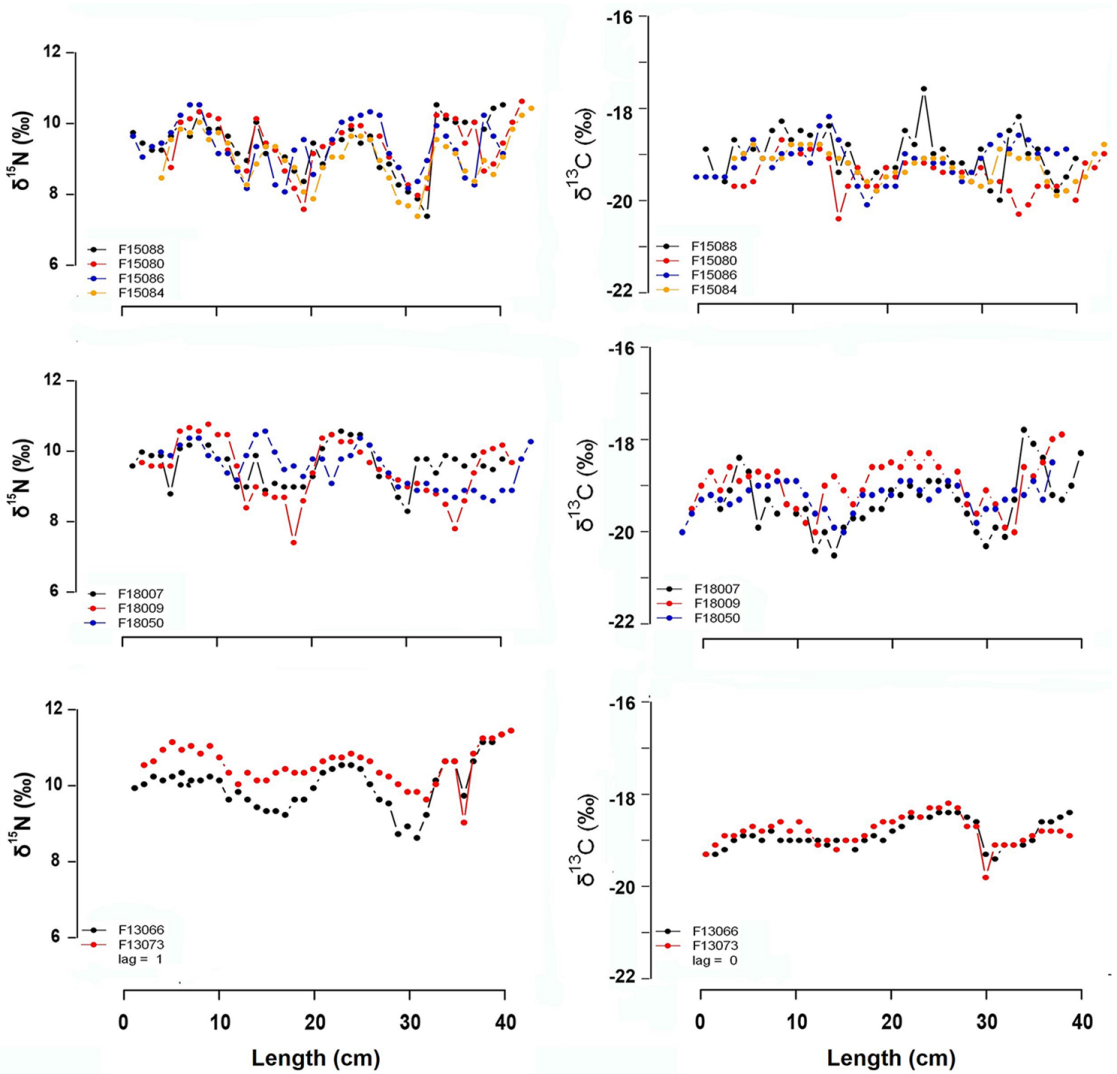
δ^15^N and δ^13^C patterns along the baleen length of the groups of individuals that were clustered together in the δ^15^N analyses and for the 2013 pair that was clustered together in both, δ^15^N and δ^13^C cluster analyses

We plotted all pairs and groups of fin whales that met the similarity requirements for one or both δ^15^N and δ^13^C values. Their δ^15^N and δ^13^C patterns along the baleen length are shown in Figs. 3 and S4. Contrasting with the variability found in all the datasets, these pairs or groups showed similar patterns along all the baleen plate growth axis, being in some cases nearly identical for both stable isotope ratios. In some cases, for the pairs of baleen whales detected through the clustering analyses, the lag calculated when analyzing δ^15^N and δ^13^C values differed up to 4 centimeters (see for example F18043 and F18054).

### Genetic analyses

DNA was extracted from 18 individuals, 9 samples from 2015 to 9 samples from 2018. Standard statistics of the microsatellite loci are summarized in Table S3. None of the microsatellite loci deviated from expected Hardy-Weinberg genotypic frequencies. The probability of identity was estimated between 1.19 × 10^− 2^ and 2.91 × 10^− 1^ while the sibling probability of identities ranged between 2.93 × 10^− 1^ and 4.90 × 10^− 1^. Two microsatellite loci presented possible presence of null allele, GATA028 and GATA43950.

The relatedness values (R) between fin whales from 2015 to 2018 (Table S4) were estimated between 0 and 0.21. However, no relationships could be assessed with a significant level of confidence (0.05 level of significance). The “specific test” was performed for the individuals with similar stable isotopes profiles, to ensure that the individuals were not related. The p-value for all the comparisons was higher than 0.05, supporting the previous results.

We would like to note that with 20 microsatellite loci, the statistical power to identify half-siblings is much lower than to identify parent-offspring pairs. In our study, the resulting low R values (below 0.15, non-significant), implies that the hypothesis that our individuals are unrelated is plausible.

## Discussion

Understanding baleen whale migratory behavior remains challenging, especially when individuals are outside the feeding grounds. In our study, we present the analysis of δ^15^N and δ^13^C values at every centimeter along the growth axis of 24 fin whale baleen plates from the western Icelandic stock. Assuming a mean growth rate of the Icelandic fin whale baleen between 16 and 20 centimeters [31, 39], the total period embraced by the study corresponds to 2–2.5 years and encompasses a minimum of two complete migratory cycles.

In most baleen whales analyzed in this study, δ^15^N and δ^13^C values showed oscillations along the baleen growth axis, with δ^15^N values displaying a notably wider range of variation. The disparities between the results of nitrogen and carbon isotope ratios can be attributed to diverse factors. In the North Atlantic, baselines exhibit more pronounced fluctuations in δ^15^N than in δ^13^C values [36], a variation pattern that coincides with our results. In baleen plates, the estimated trophic increase is similar for δ^15^N (2.77‰) and δ^13^C values (2.26‰) [35], although δ^15^N values in other tissues and mammals usually display a greater increase in each trophic level than δ^13^C values do [34, 35]. δ^15^N values can be also influenced by physiological factors such as nutritional condition or fasting, although impact of the latter on baleen whales remains the subject of discussion [31, 32]. δ^13^C values are affected by proximity to coastal habitats and, because fin whales are primarily distributed in offshore waters [17], we did not anticipate substantial variation in the δ^13^C oscillations in their baleen. All of the above would lead us to expect that the variation in the values of δ^15^N would be greater than in those of δ^13^C, as we have found.

Whatever the case, variability of δ^15^N and δ^13^C oscillations between individuals was large, which explains why the different cluster methodologies failed to identify a common structure within the Icelandic population. Compared to other baleen whales whose migratory behavior is better known, such as humpback whales, the migration of the *Balaenoptera* species seems to be less regular and predictable. This is particularly true in the North Atlantic, where individuals appear to disperse in offshore waters during winter, occupying southern breeding grounds that may potentially span hundreds of kilometers [9, 17, 57, 58]. Dispersal during winter may provide some advantages, such as a better chance of feeding opportunistically during the breeding season and during migration [6, 59], but it may also present some disadvantages, such as hampering communication and coordination between conspecifics.

Surprisingly, despite the overall variability in δ^15^N and δ^13^C values oscillations that was observed among individuals, clustering analyses highlighted similarities across different individuals. Several of them, with no kinship, exhibited analogous patterns along the growth axis of the baleen plate for both δ^15^N and/or δ^13^C values during the two consecutive years of isotopic record (Fig. 3, Figure S4). For some of these pairs or trios, clustering analyses failed to obtain the same results when using δ^15^N or δ^13^C values. While this can be explained by the aforementioned metabolic factors influencing δ^15^N and δ^13^C values, in some cases the absence of consistency in the clustering analyses may also be attributed to technical limitations of the statistical analyses. For example, differences in the overall variability and similarity of oscillations considered in an analysis have a strong effect in the clustering, and this variability differs between δ^15^N and δ^13^C values. Also, in certain baleen whales, both stable isotope ratios demonstrated analogous patterns but diverged at some specific centimeters along the baleen plate record for one of the two stable isotope ratios (for example δ^13^C value for the pair F15080-F15088). All these factors may lead to dissimilar results during the clustering analyses even if oscillations of both individuals are similar and animals share common migratory patterns (Figure S4).

We suggest two different and mutually compatible explanations for our results: (i) existence of long-term relationship between the involved individuals, and/or (ii) occurrence of specific migratory regimes that, being particularly beneficial, are shared by some individuals of the same stock. The fin whale, as most other balaenopterids, is believed to be non-gregarious during winter [17]. Conversely, large aggregations of fin whales (of up to dozens of individuals) may be seen in the feeding grounds, although mainly singles or pairs are observed [23, 60]. Indeed, the larger aggregations are likely ephemeral and driven by the aggregation of their prey. In some Balaenopterids, smaller groups, usually composed of 2–3 individuals, seem to be more stable during the feeding season [14]. For example, humpback whales show multi-year stable associations, mostly involving pairs of individuals, while they are at the feeding grounds, a behaviour apparently linked to cooperative feeding [14–16]. However, it is considered improbable that long-term relationships among humpback whales are maintained throughout the year, because the associations in the feeding grounds appear to be different than those in the breeding grounds [15].

However, fin whales seem to depart from this behavior. While at the feeding grounds, they do not often engage in cooperative feeding and their migratory and breeding behavior appears very different than that of humpback whales. However, this does not necessarily preclude the occurrence of association because in other groups of animals collective migratory strategies may evolve even when social encounters are rare, demonstrating that social interactions still may play an important role in sparse organisms [61].

Fin whales, and other baleen whales such as blue whales, produce low-frequency pulses which can propagate over long ranges in the ocean [58]. It seems that the main reason for producing these sounds is to attract females in the feeding grounds or to locate prey patches [62, 63]. However, the sounds may also be used during migration and throughout the winter season to coordinate the behavior between individuals, as has been observed to happen in blue whales [64]. Fin whales may be able to stay in acoustic contact over hundreds of kilometers, and can thus migrate as a group even when individuals are dispersed over a wide breeding area and apparently travel alone [4]. This may be a potential explanation to the sharp similitude between the δ^15^N and δ^13^C oscillations observed between the baleen of some individuals.

We did not find any specific genetic relationship between individuals that showed the most similar migratory patterns, so the associations cannot be linked to matrilineal or other consanguinity ties within the collective of whales exploited off western Iceland [26]. It has been adduced that long-term association between non-related individuals could provide an opportunity to modify and improve the vertically learned migratory routes and then transfer them to offspring [65, 66]. As a result, vertical and horizontal transference of knowledge on migration, breeding and feeding grounds can create a collective memory [4, 7]. As it has been shown in other animal groups, adopting the parental migratory route in adult life, rather than dispersing randomly, may increase an individual’s reproductive success because that strategy has already been proven to allow successful breeding [67]. Such behavior could have relevant consequences for the long-term stability of the population because, if all individuals with that knowledge are extirpated from the population, this information may be lost [7].

However, the similarities observed in the baleen δ^15^N and δ^13^C oscillations may also be due to factors other than stable associations of individuals. Some specific migratory regimes may be more beneficial, and then be shared between individuals without the need of being physically or acoustically associated. This explanation could be especially true for the groups detected during the clustering analyses (Figure S4) or for pairs with similar δ^13^C values but variations in their δ^15^N values (see for example F18050 and F18030, or F18044 and F18003, Figure S3). Considering that breeding grounds could extend over hundreds of kilometers, fin whales inhabiting the same regions may engage in distinct feeding behaviors, thereby influencing their δ^15^N values. During the summer months, fin whales in Icelandic waters predominantly feed on krill [68, 69] but, during the winter season, they appear to transition to a more generalist diet that includes higher trophic level prey such as schooling fish [68, 70, 71]. This dietary shift and enhanced opportunistic foraging may lead to high variations in their δ^15^N values [71]. In addition, physiological condition and reproductive status may also influence δ^15^N values [31, 35]. In our results, pairs consistently clustered together when analyzing δ^15^N oscillations were always from the same sex. This observation suggests that these individuals may not only share similar migratory patterns and dietary compositions, but also be subject to analogous physiological constraints.

In any case, the putative trios or pairs detected may reflect some degree of internal structure in the fin whales visiting western Icelandic waters in the summer. Genetic analyses have shown that fin whales from the same feeding ground use different breeding grounds [51], and this may be the case for western Iceland, where the community of whales visiting the region seems to be composed of a mixture of several breeding populations or sub-populations [19]. Specifically, the population summering off Iceland appears to be composed mainly of individuals wintering in the central Atlantic, including the Azores region [6, 19]. In addition, recent studies suggest that fin whales sampled in the Azores during spring had been previously feeding off northwestern Spain during the winter, supporting a sequential occupation of the latter area by fin whales from different feeding grounds [59, 72]. Whales clustered or paired together seem to share the same migratory regime and destinations, and thus would have a common and distinctive isotopic signal without having necessarily to coordinate their migrations. Alternatively, the grouping may reflect spatial and/or temporal segregation according to age and sex within the population, as it has been shown for fin whales in this area and elsewhere [73, 74]. This latter option may include the possibility of remaining in high northern latitudes even in the winter [75].

The stable isotope ratios alone cannot discriminate between the various explanations proposed here, and other approaches and methodologies are required to resolve this issue. Clarification is likely to come with the combined use of satellite tracking [6, 75], advanced genetic analyses [51, 76], in-depth study of vocalizations [77, 78], or the application of other chemical and biochemical tracers, such as fatty acids or indicative natural or xenobiotic markers [79–82].

In summary, cluster analyses provide a useful exploratory tool for discerning common patterns in the isotopic oscillations, particularly when dealing with a large number of samples exhibiting significant variability. Combination of δ^13^C and δ^15^N oscillation patterns in the baleen of fin whales feeding off west Iceland reflect an offshore dispersion in the winter breeding grounds. However, and despite such variability, some individuals presented similar stable isotope ratios along two consecutive years, which suggests either potential long-term associations between the involved individuals and/or common migratory regimes, reflecting some degree of internal population structure in the western Iceland fin whale stock.

### Electronic supplementary material

Below is the link to the electronic supplementary material.

Supplementary Material 1

## Data Availability

Raw data will be made available on request to Alex Aguilar or Asunción Borrell.
