# Supplementary material for: Order within chaos: potential migratory strategies and individual associations in fin whales feeding off Iceland
Source: Mov Ecol. 2024 May 9;12:36. doi: 10.1186/s40462-024-00474-w (PMC11080271; doi:10.1186/s40462-024-00474-w)
Supplement: Supplementary file 1 — Supplementary Material 1 [file 40462_2024_474_MOESM1_ESM.docx]

***SUPPLEMENTARY MATERIALS: Order within chaos: potential migratory strategies and individual associations in fin whales feeding off Iceland***

***Table S1****: Mean and standard deviations of δ15N and δ13C values for each individual. Sex and date of capture of each animal is also indicated.*

| **Individual** | **Sex** | **Day of capture** | **Year** | **Mean dN** | **SD dN** | **Mean dC** | **SD dC** |
| --- | --- | --- | --- | --- | --- | --- | --- |
| F13065 | Female | 23/07 | 2013 | 9.27 | 0.65 | -19.25 | 0.34 |
| F13066 | Male | 23/07 | 2013 | 9.96 | 0.62 | -18.87 | 0.29 |
| F13068 | Female | 23/07 | 2013 | 10.32 | 1.04 | -19.62 | 0.26 |
| F13073 | Male | 27/07 | 2013 | 10.50 | 0.49 | -18.81 | 0.31 |
| F13076 | Male | 29/07 | 2013 | 11.09 | 1.07 | -19.36 | 0.29 |
| F15078 | Male | 07/08 | 2015 | 9.38 | 0.70 | -19.01 | 0.47 |
| F15097 | Female | 23/08 | 2015 | 9.68 | 1.11 | -18.81 | 0.36 |
| F15086 | Female | 15/08 | 2015 | 9.28 | 0.72 | -19.14 | 0.40 |
| F15083 | Female | 10/08 | 2015 | 8.91 | 1.21 | -19.04 | 0.46 |
| F15080 | Male | 08/08 | 2015 | 9.33 | 9.76 | -19.45 | 0.39 |
| F15079 | Female | 08/08 | 2015 | 8.92 | 1.04 | -19.38 | 0.34 |
| F15084 | Female | 10/08 | 2015 | 8.98 | 0.73 | -19.23 | 0.32 |
| F15087 | Male | 15/08 | 2015 | 9.29 | 1.07 | -19.10 | 0.49 |
| F15088 | Male | 15/08 | 2015 | 9.36 | 0.74 | -19.02 | 0.50 |
| F18044 | Female | 20/07 | 2018 | 10.91 | 1.62 | -19.68 | 0.57 |
| F18043 | Female | 20/07 | 2018 | 8.79 | 1.18 | -19.07 | 0.34 |
| F18038 | Female | 18/07 | 2018 | 10.53 | 1.21 | -19.43 | 0.27 |
| F18050 | Female | 23/07 | 2018 | 9.54 | 0.58 | -19.25 | 0.33 |
| F18051 | Female | 23/07 | 2018 | 10.47 | 1.17 | -19.30 | 0.39 |
| F18030 | Female | 14/07 | 2018 | 10.21 | 1.38 | -19.31 | 0.35 |
| F18054 | Female | 25/07 | 2018 | 9.00 | 0.81 | -19.06 | 0.36 |
| F18007 | Male | 28/06 | 2018 | 9.65 | 0.56 | -19.33 | 0.61 |
| F18003 | Male | 24/06 | 2018 | 10.54 | 0.99 | -19.82 | 0.39 |
| F18009 | Male | 30/06 | 2018 | 9.50 | 0.83 | -18.94 | 0.51 |

***Table S2****: Results from the adapted version of the Manhattan distance. For each individual, the corresponding pairs that showed lowest dissimilarity values are shown. This analysis was performed without extracting any datapoint from the dataset.*

| **Individual** | **Best dN** | **Second Best dN** | **Best dC** | **Second Best dC** |
| --- | --- | --- | --- | --- |
| **F13065** | F13066 | F13073 | F13076 | F13066 |
| **F13066** | F13073 | F13065 | F13073 | F13065 |
| **F13068** | F13066 | F13073 | F13076 | F13065 |
| **F13073** | F13066 | F13068 | F13066 | F13065 |
| **F13076** | F13073 | F13068 | F13068 | F13065 |
| **F15078** | F15088 | F15086 | F15086 | F15083 |
| **F15097** | F15086 | F15087 | F15083 | F15078 |
| **F15086** | F15080 | F15084 | F15083 | F15078 |
| **F15083** | F15079 | F15084 | F15086 | F15078 |
| **F15080** | F15088 | F15086 | F15084 | F15079 |
| **F15079** | F15084 | F15083 | F15087 | F15084 |
| **F15084** | F15080 | F15086 | F15086 | F15080 |
| **F15087** | F15088 | F15080 | F15079 | F15086 |
| **F15088** | F15080 | F15078 | F15084 | F15083 |
| **F18044** | F18051 | F18030 | F18038 | F18003 |
| **F18043** | F18054 | F18009 | F18054 | F18009 |
| **F18038** | F18007 | F18030 | F18050 | F18051 |
| **F18050** | F18009 | F18007 | F18030 | F18038 |
| **F18051** | F18003 | F18044 | F18038 | F18030 |
| **F18030** | F18009 | F18050 | F18050 | F18038 |
| **F18054** | F18043 | F18050 | F18043 | F18050 |
| **F18007** | F18009 | F18050 | F18038 | F18030 |
| **F18003** | F18051 | F18038 | F18038 | F18044 |
| **F18009** | F18007 | F18050 | F18043 | F18050 |

***Table S3.*** *Summary results for the 20 microsatellite loci genotyped in 18 individuals. Note. Na denote the number of alleles; N, the number of individuals typed; Ho and He, the observed and expected heterozygosity; PIth, probability of identity and Pisib, sibling probability of identity*

| **Locus** | **Na** | **Ho** | **He** | **PIth** | **PIsib** |
| --- | --- | --- | --- | --- | --- |
| AC087 | 3 | 0,5 | 0,64 | 2,09E-01 | 4,84E-01 |
| CA234 | 7 | 0,83 | 0,81 | 6,42E-02 | 3,63E-01 |
| EV001 | 9 | 1 | 0,82 | 5,47E-02 | 3,53E-01 |
| EV037 | 9 | 0,78 | 0,8 | 6,34E-02 | 3,67E-01 |
| EV094 | 14 | 1 | 0,9 | 1,86E-02 | 3,05E-01 |
| GATA028 | 16 | 0,83 | 0,92 | 1,19E-02 | 2,93E-01 |
| GATA098 | 6 | 0,83 | 0,78 | 8,17E-02 | 3,78E-01 |
| GATA417 | 12 | 0,94 | 0,88 | 2,43E-02 | 3,15E-01 |
| GATA43950 | 7 | 0,5 | 0,61 | 1,80E-01 | 4,90E-01 |
| GATA52422 | 11 | 0,72 | 0,83 | 4,87E-02 | 3,48E-01 |
| GATA5947654 | 7 | 0,72 | 0,76 | 9,03E-02 | 3,91E-01 |
| GATA6063318 | 9 | 0,67 | 0,64 | 1,57E-01 | 4,68E-01 |
| GATA91083 | 6 | 0,72 | 0,71 | 1,29E-01 | 4,27E-01 |
| GT011 | 7 | 0,89 | 0,85 | 4,30E-02 | 3,38E-01 |
| GT023 | 8 | 0,72 | 0,78 | 8,01E-02 | 3,81E-01 |
| GT211 | 7 | 0,83 | 0,78 | 7,78E-02 | 3,78E-01 |
| GT271 | 6 | 0,61 | 0,68 | 1,57E-01 | 4,49E-01 |
| GT310 | 6 | 0,83 | 0,64 | 1,75E-01 | 4,73E-01 |
| GT575 | 7 | 0,72 | 0,75 | 9,00E-02 | 3,96E-01 |
| TAA023 | 5 | 0,72 | 0,71 | 1,32E-01 | 4,28E-01 |

***Table S4:*** *ML-Relate output* *matrix of maximum likelihood relatedness (R) between fin whales from 2015 and 2018. All the resulting R values are low (below 0.15, non-significant)*

|  | **15078** | **15079** | **15080** | **15083** | **15084** | **15086** | **15087** | **15088** | **15097** | **18003** | **18007** | **18009** | **18038** | **18043** | **18044** | **18050** | **18051** | **18054** |
| --- | --- | --- | --- | --- | --- | --- | --- | --- | --- | --- | --- | --- | --- | --- | --- | --- | --- | --- |
| **15078** | 1.000 |  |  |  |  |  |  |  |  |  |  |  |  |  |  |  |  |  |
| **15079** | 0.000 | 1.000 |  |  |  |  |  |  |  |  |  |  |  |  |  |  |  |  |
| **15080** | 0.000 | 0.120 | 1.000 |  |  |  |  |  |  |  |  |  |  |  |  |  |  |  |
| **15083** | 0.000 | 0.000 | 0.000 | 1.000 |  |  |  |  |  |  |  |  |  |  |  |  |  |  |
| **15084** | 0.000 | 0.000 | 0.000 | 0.000 | 1.000 |  |  |  |  |  |  |  |  |  |  |  |  |  |
| **15086** | 0.000 | 0.000 | 0.000 | 0.000 | 0.000 | 1.000 |  |  |  |  |  |  |  |  |  |  |  |  |
| **15087** | 0.000 | 0.000 | 0.050 | 0.000 | 0.020 | 0.000 | 1.000 |  |  |  |  |  |  |  |  |  |  |  |
| **15088** | 0.000 | 0.060 | 0.010 | 0.000 | 0.000 | 0.000 | 0.000 | 1.000 |  |  |  |  |  |  |  |  |  |  |
| **15097** | 0.000 | 0.020 | 0.000 | 0.160 | 0.000 | 0.020 | 0.030 | 0.000 | 1.000 |  |  |  |  |  |  |  |  |  |
| **18003** | 0.020 | 0.000 | 0.090 | 0.000 | 0.010 | 0.000 | 0.090 | 0.000 | 0.000 | 1.000 |  |  |  |  |  |  |  |  |
| **18007** | 0.000 | 0.000 | 0.000 | 0.000 | 0.000 | 0.000 | 0.000 | 0.080 | 0.000 | 0.000 | 1.000 |  |  |  |  |  |  |  |
| **18009** | 0.000 | 0.000 | 0.000 | 0.120 | 0.000 | 0.000 | 0.000 | 0.020 | 0.040 | 0.000 | 0.050 | 1.000 |  |  |  |  |  |  |
| **18038** | 0.000 | 0.000 | 0.020 | 0.000 | 0.000 | 0.000 | 0.000 | 0.030 | 0.000 | 0.030 | 0.020 | 0.170 | 1.000 |  |  |  |  |  |
| **18043** | 0.000 | 0.000 | 0.020 | 0.000 | 0.000 | 0.000 | 0.020 | 0.000 | 0.000 | 0.000 | 0.000 | 0.000 | 0.000 | 1.000 |  |  |  |  |
| **18044** | 0.000 | 0.040 | 0.070 | 0.000 | 0.060 | 0.000 | 0.000 | 0.000 | 0.000 | 0.130 | 0.040 | 0.000 | 0.000 | 0.130 | 1.000 |  |  |  |
| **18050** | 0.000 | 0.000 | 0.000 | 0.010 | 0.030 | 0.000 | 0.000 | 0.030 | 0.070 | 0.000 | 0.000 | 0.000 | 0.000 | 0.000 | 0.000 | 1.000 |  |  |
| **18051** | 0.000 | 0.000 | 0.000 | 0.050 | 0.000 | 0.020 | 0.000 | 0.000 | 0.010 | 0.090 | 0.000 | 0.000 | 0.040 | 0.000 | 0.120 | 0.000 | 1.000 |  |
| **18054** | 0.070 | 0.050 | 0.000 | 0.100 | 0.000 | 0.000 | 0.130 | 0.000 | 0.000 | 0.000 | 0.000 | 0.000 | 0.020 | 0.020 | 0.000 | 0.000 | 0.000 | 1.000 |

*
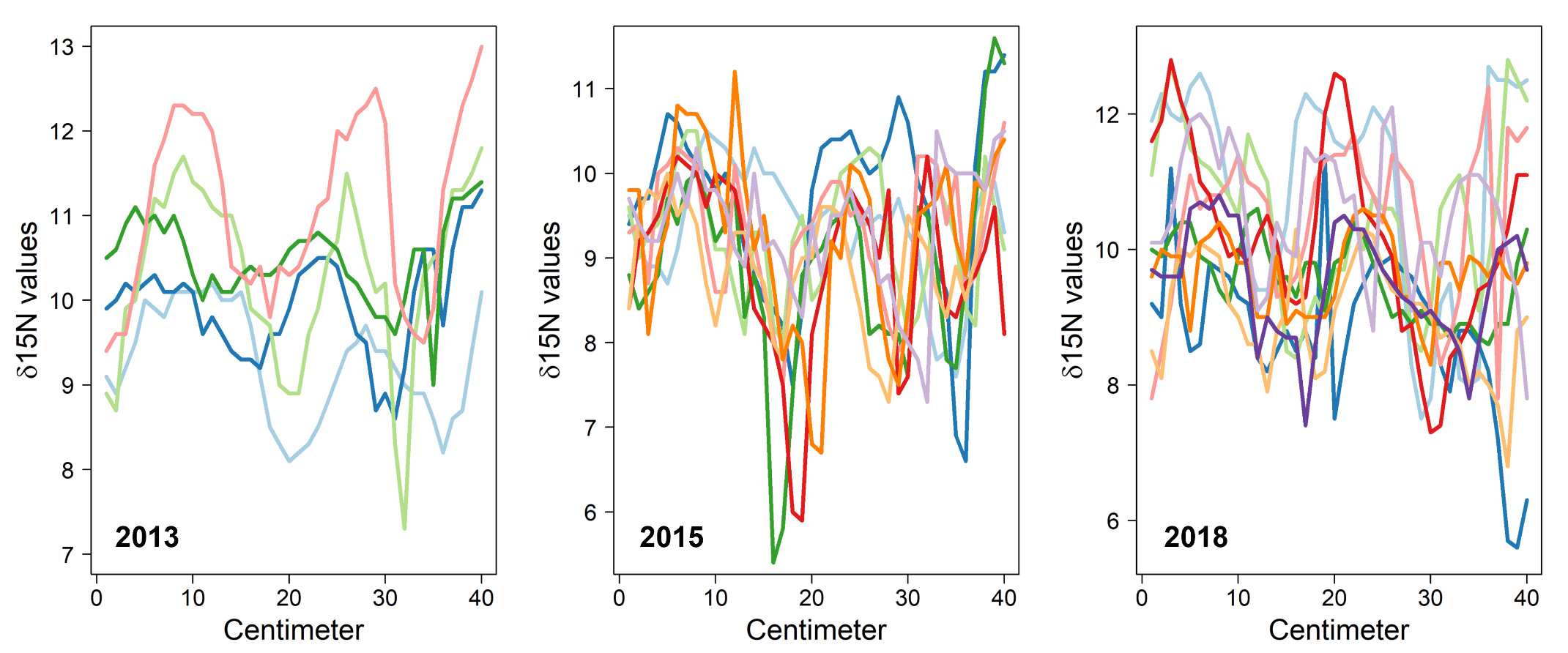
*

***Figure S1****: Raw data of δ^15^N patterns along the baleen length of fin whales from 2013 (left), 2015 (mid) and 2018 (right). No correction or alignment was performed before plotting the raw data. The initial point corresponds to the first centimeter that was sampled inside the gum, which varied from one individual to another, depending on the size of the baleen plate and morphology.*

*
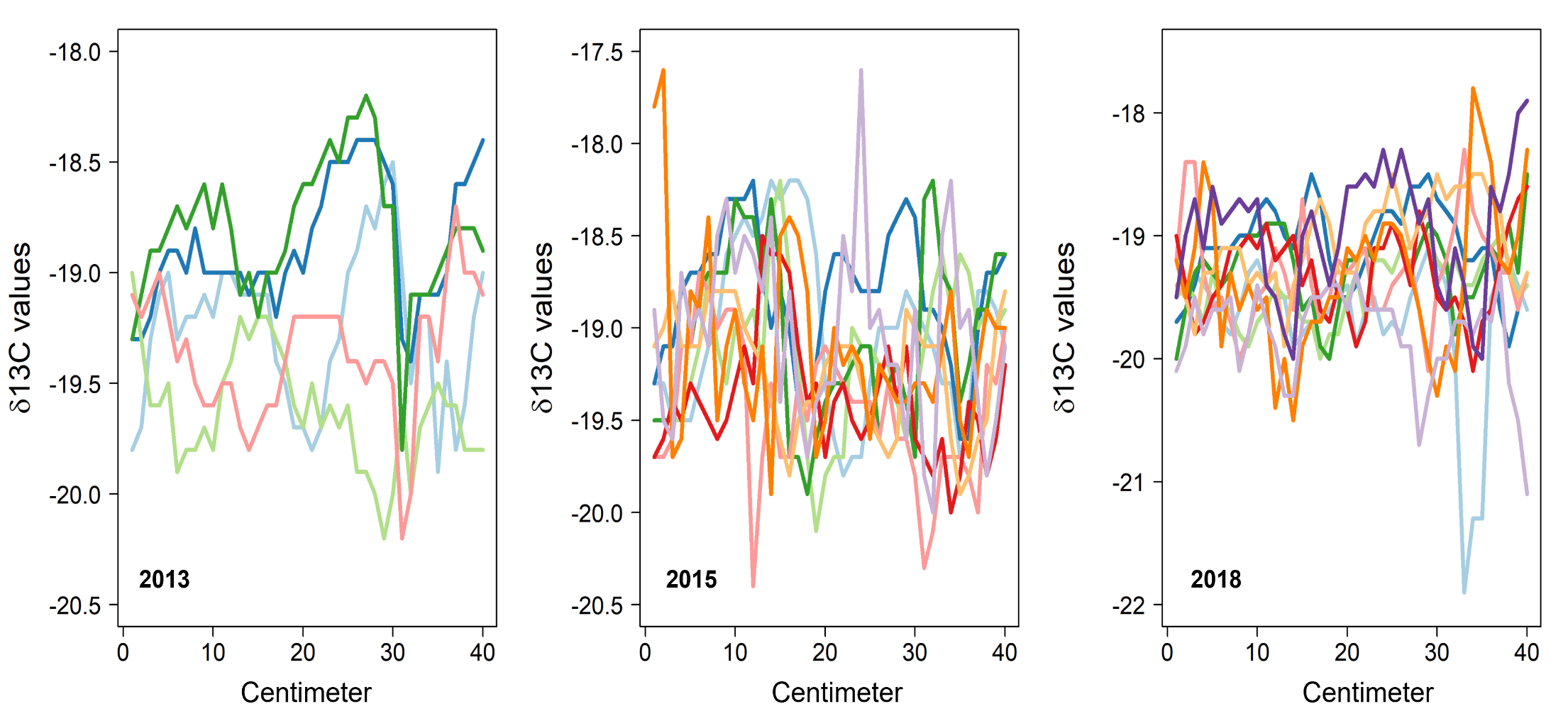
*

***Figure S2****: Raw data of δ^13^C patterns along the baleen length of fin whales from 2013 (left), 2015 (mid) and 2018 (right). No correction or alignment was performed before plotting the raw data. The initial point corresponds to the first centimeter that was sampled inside the gum, which varied from one individual to another, depending on the size of the baleen plate and morphology.*

*
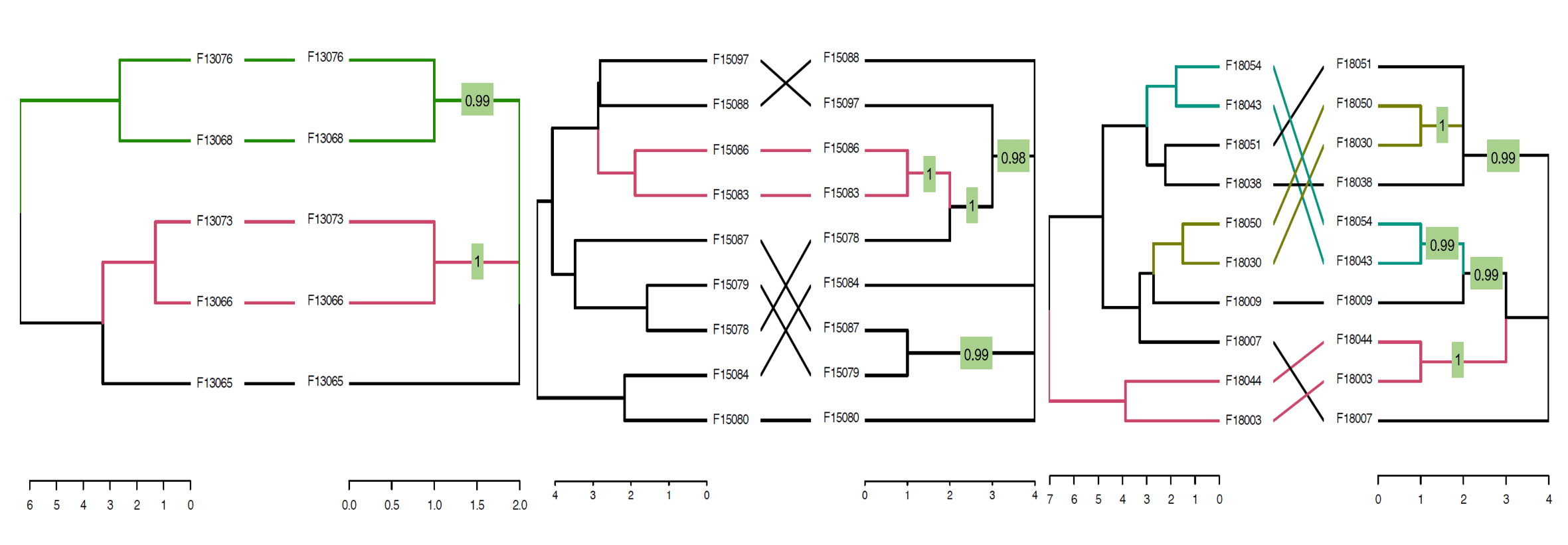
*

***Figure S3****: Dendrograms showing the results for 2013 (left), 2015 (middle) and 2018 (right) datasets for δ^13^C values. Results from the DTW clustering are presented at the left dendrogram of each panel, and results from the CFF clustering are presented at the right. Colored lines show common branches and groups for both methodologies. Red squares show groups that are composed by the same individuals, although the internal structure is not maintained. Finally, in the CFF clustering, branches in which p>0.95 are indicated inside green boxes.*

***
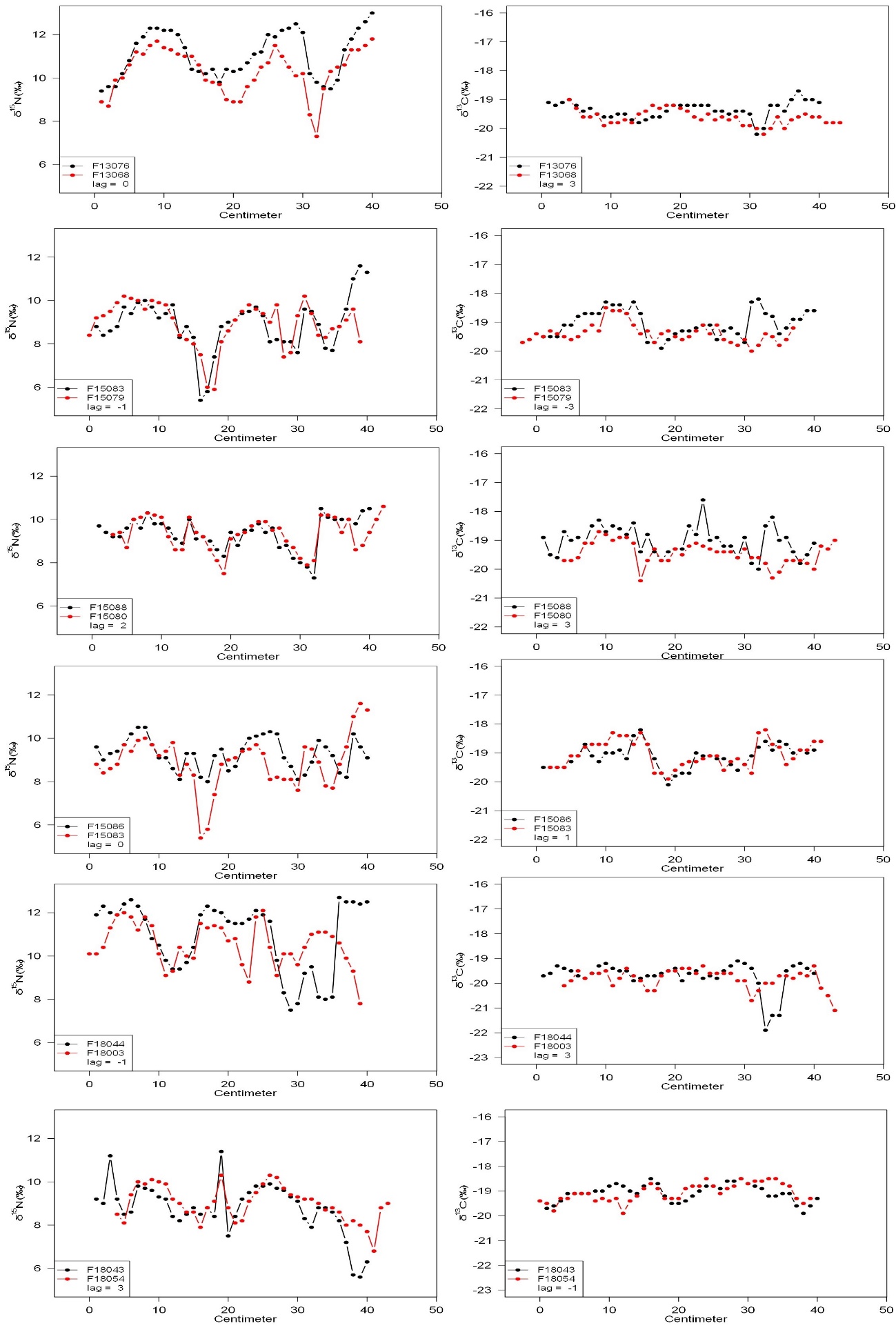
***

***Figure S4****: Pairs grouped during the cluster analyses with δ^15^N and δ^13^C. In all cases, we show their δ^15^N (left) and δ^13^C (right) values with the optimal lag determined during clustering.*

***Figure S4 (continuation)****: Pairs grouped during the cluster analyses with δ^15^N and δ^13^C. In all cases, we show their δ^15^N (left) and δ^13^C (right) values with the optimal lag determined during clustering*
